# The human ABC transporter pseudogene family: Evidence for transcription and gene-pseudogene interference

**DOI:** 10.1186/1471-2164-9-165

**Published:** 2008-04-11

**Authors:** Armin P Piehler, Marit Hellum, Jürgen J Wenzel, Ellen Kaminski, Kari Bente Foss Haug, Peter Kierulf, Wolfgang E Kaminski

**Affiliations:** 1 Department of Clinical Chemistry, Ulleval University Hospital, 0407 Oslo, Norway; 2 Institute for Medical Microbiology and Hygiene, University of Regensburg, 93053 Regensburg, Germany; 3 Department of Hygiene and Medical Microbiology, University of Heidelberg, 69120 Heidelberg, Germany; 4 Institute for Clinical Chemistry, University of Heidelberg, 68167 Mannheim, Germany

## Abstract

**Background:**

Pseudogenes are an integral component of the human genome. Little attention, however, has so far been paid to the phenomenon that some pseudogenes are transcriptionally active. Recently, we demonstrated that the human ortholog of the rodent testis-specific ATP-binding cassette (ABC) transporter Abca17 is a ubiquitously transcribed pseudogene (*ABCA17P*). The aim of the present study was to establish a complete inventory of all ABC transporter pseudogenes in the human genome and to identify transcriptionally active ABC transporter pseudogenes. Moreover, we tested the hypothesis that a regulatory interdependency exists between ABC transporter pseudogenes and their parental protein coding equivalents.

**Results:**

Systematic bioinformatic analysis revealed the existence of 22 ABC transporter pseudogenes within the human genome. We identified two clusters on chromosomes 15 and 16, respectively, which harbor almost half of all pseudogenes (n = 10). Available information from EST and mRNA databases and RT-PCR expression profiling indicate that a large portion of the ABC transporter pseudogenes (45%, n = 10) are transcriptionally active and some of them are expressed as alternative splice variants. We demonstrate that both pseudogenes of the pseudoxanthoma elasticum gene *ABCC6*, *ABCC6P1 *and *ABCC6P2*, are transcribed. *ABCC6P1 *and *ABCC6 *possess near-identical promoter sequences and their tissue-specific expression profiles are strikingly similar raising the possibility that they form a gene-pseudogene dual transcription unit. Intriguingly, targeted knockdown of the transcribed pseudogene *ABCC6P1 *resulted in a significant reduction of *ABCC6 *mRNA expression levels.

**Conclusion:**

The human genome contains a surprisingly small number of ABC transporter pseudogenes relative to other known gene families. They are unevenly distributed across the chromosomes. Importantly, a significant portion of the ABC transporter pseudogenes is transcriptionally active. The downregulation of *ABCC6 *mRNA levels by targeted suppression of the expression of its pseudogene *ABCC6P1 *provides evidence, for the first time, for a regulatory interdependence of a transcribed pseudogene and its protein coding counterpart in the human genome.

## Background

Human ATP-binding cassette (ABC) transporters form a large family of structurally highly related membrane transport proteins. Based on sequence similarities, the human ABC transporter family is categorized into seven subfamilies, denoted ABC A-G, and consists of 48 members. ABC transporters translocate a wide range of substrates across cellular membranes, e.g. lipids, sugars, peptides, ions and xenobiotics such as anticancer drugs [1,2]. Defective ABC transporters cause monogenetic disorders and contribute to the development of complex diseases [3]. The physiological importance of ABC proteins is highlighted by the fact that mutations in 14 human ABC transporter genes have thus far been linked to a spectrum of unrelated hereditary diseases [2].

Beside protein coding genes for ABC transporters, it has been shown that the human genome contains several ABC transporter pseudogenes [4]. However, the exact number of ABC transporter pseudogenes present in the human genome is still unclear. Generally, pseudogenes are defined as non-functional sequences that are present in the genome with high similarity to one or more paralogous genes. This lack of function is a result of either failure of transcription or translation, or production of a protein that differs in function from the protein encoded by the functional gene [5]. Pseudogenes are thought to arise either by gene duplication and subsequent degeneration of the duplicated gene or by retroposition of a reversely transcribed mRNA of the paralogous gene into the genome. The latter are intronless and referred to as "processed" pseudogenes [6].

During the past years, we and others provided evidence for the existence of ABC transporter pseudogenes which are transcriptionally active [7-9]. For example, we recently demonstrated that the human ortholog of the rodent testis-specific ABC transporter Abca17, a protein coding gene, is a pseudogene (*ABCA17P*) which is transcribed into up to 20 alternative splice variants [9]. These results raise the intriguing possibility that ABC transporter pseudogene transcripts may serve biological functions other than providing the genetic blueprints for protein biosynthesis.

In this study, we applied a comprehensive bioinformatic approach to identify all ABC transporter pseudogenes in the human genome and systematically searched for evidence of transcribed pseudogenes. We identified a total of 22 pseudogenes within the ABC transporter family with a significant subfraction (n = 10) exhibiting transcriptional activity. We find evidence for transcription of the two previously known *ABCC6 *pseudogenes *ABCC6P1 *and *ABCC6P2*, respectively, and report their gene structures. Moreover, we demonstrate, for the first time, a regulatory interdependency between *ABCC6P1*, and its translated counterpart, the pseudoxanthoma elasticum gene *ABCC6*.

## Results

### The human genome contains 22 ABC transporter pseudogenes

**Figure 1:**
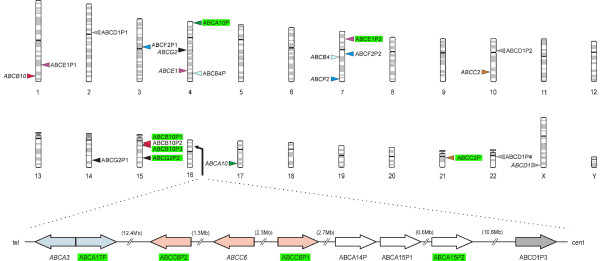
**Chromosomal distribution of human ABC transporter pseudogenes and protein-coding genes**. Pseudogenes of the human ABC transporter family are widely distributed throughout the human genome. Shown are the chromosomal localizations (colored arrows) of ABC transporter pseudogenes (right-hand side of each chromosome) and their parental protein-coding genes (left) in the human genome. Pairs of parental genes and pseudogenes are represented by the same arrow color code. Transcribed pseudogenes are green shaded. A close-up view of the ABC transporter pseudogene cluster (30 Mb) on chromosome 16p is shown at the bottom. Arrows indicate the 5'-> 3' orientation of the pseudogenes on chr. 16p and distances between adjacent pseudogenes are detailed (Mb). *ABCA14P*, *ABCA15P1 *and *ABCA15P2*, respectively, represent pseudogenes for which functional counterparts have only been reported in rodents (Abca14, Abca15). The ideogram was generated using the ColoredChromosomes software [41].

To identify homologs of human ABC transporters, we performed a genome-wide search based on the sequences of the known 48 human and the five murine ABC transporter genes, for which no orthologous counterpart as yet have been documented in the human genome (*Abca14*-*Abca17*,*Abcg3*). The strategy relied on a BLAST homology search (cut-off E-value: 0.001) in which the ABC transporter nucleotide reference sequences were used as query sequences. These were compared with a version of the human genome (assembly March 2006, NCBI Build 36, hg18) in which the known exons of the ABC transporter query sequences were masked. Our search algorithm resulted in 3,322,841 primary hits. Detailed sequence analyses revealed that a surprisingly large fraction (33/48, 69%) of the known human ABC transporter cDNAs contain repetitive elements (Additional File 1). We then excluded all hits that were attributable to repetitive elements and assorted the remaining hits (n = 174) according to their chromosomal position. Neighbouring hits were merged to a single pseudogene candidate when they (i) displayed highest similarity to the same protein coding ABC transporter gene, (ii) were localized at a distance < 0.1 Mb apart from each other and (iii) were oriented in the same 5'→ 3' order as the matching protein coding ABC transporter nucleotide sequence. In those cases where a neighbouring hit was absent, a flanking 0.1 Mb genomic region was aligned with the respective ABC transporter nucleotide sequence using the more rigorous alignment tool "matcher" from the EMBOSS package. This approach warrants that more distantly related ABC transporter pseudogenes are overlooked at a minimal rate. Hits with > 70% identity across at least one complete exon of the respective ABC transporter nucleotide sequence, were considered homologous and evaluated by the above criteria (i-iii). Merged hits were regarded as pseudogenes when they encompassed at least two homologous exons of the protein-coding gene and had defective reading frames which preclude translation into polypeptides that meet the minimum structural requirements for functional ABC full-size or half-size transporters. Using this strategy, we identified a total of 22 loci in the human genome that represent degenerated copies of protein coding ABC transporter genes. They show > 70% similarity to the cDNA of their protein coding counterparts across a range of 159 – 3754 bp and contain the homologs of two up to 19 exons of the respective parental gene. The ABC transporter pseudogenes we identified could be assigned to all seven subfamilies, ABC A- ABC G (Table 1). We found that, within the genome, they are routinely present on chromosomes which also harbor protein coding ABC genes (Chrs. 1, 2, 3, 4, 7, 10, 14, 16, 21) with the exception of chrs. 15 and 22, respectively, from which protein coding ABC transporter genes are absent (Figure 1). Of note, half of the 22 ABC transporter pseudogenes are localized on chromosomes 15 (n = 4) and 16 (n = 7), respectively. Intriguingly, the seven pseudogenes on the short arm of chr. 16 (16p11.2-p13.3) are clustered within a segment of 30 Mb in the order *tel*-*ABCA17P *– *ABCC6P2 *– *ABCC6P1 *– *ABCA14P *– *ABCA15P1 *– *ABCA15P2 *– *ABCD1P3*-*cer*.

**Table 1:** ABC transporter pseudogenes in the human genome

Parental gene	ψ-gene symbol	Chromosomal band	Chromosomal position*	Processed	Evidence for transcription	Alternatively spliced transcripts	Accession number**
*ABCA10*	*ABCA10P (ABCA11)*	4p16.3	408750–411669	+	+	3	AK024359
*ABCA3/Abca17*	*ABCA17P*	16p13.3	2330924–2416701	-	+	20	DQ266102
*ABCB4*	*ABCB4P*	4q32.1	158955613–158956084	+	-	-	
*ABCB10*	*ABCB10P1*	15q11.2	20240777–20244535	+	+	nd	DB511925
	*ABCB10P2*	15q13.1	26321621–26325378	+	-	-	
	*ABCB10P3*	15q13.1	26552813–26550744	+	+	nd	BP260092
*ABCC2*	*ABCC2P (ABCC13)*	21q11.2	14568083–14593875	-	+	-	AY344117
*ABCC6*	*ABCC6P1*	16p12.3	18490076–18511541	-	+	-	BC075833
	*ABCC6P2*	16p13.11	14822152–14826050	-	+	-	BC015978
*ABCD1*	*ABCD1P1*	2p11.1	91392363–91396515	-	-	-	
	*ABCD1P2*	10p11.1	38934556–38938703	-	-	-	
	*ABCD1P3*	16p11.2	32392919–32397074	-	-	-	
	*ABCD1P4*	22q11.1	15248418–15252575	-	-	-	
*ABCE1*	*ABCE1P1*	1q31.2	190481983–190483713	+	-	-	
	*ABCE1P2*	7p15.3	23566974–23570537	+	+	nd	AY962469
*ABCF2*	*ABCF2P1*	3p11.2	88448934–88451415	+	-	-	
	*ABCF2P2*	7q11.2	71206102–71208540	-	-	-	
*ABCG2*	*ABCG2P1*	14q24.3	76895512–76896054	+	-	-	
	*ABCG2P2*	15q23	67101380–67103614	+	+	nd	CR610432
*Abca14*	*ABCA14P*	16p12.2	21210041–21219286	-	-	-	
*Abca15*	*ABCA15P1*	16p12.2	21257276–21257846	-	-	-	
	*ABCA15P2*	16p12.1	21857685–21863768	-	+	2	DR731461

*Chromosome positions refer to the human genome NCBI Build 36 (hg18, assembly March 2006).

**Accession numbers refer to identified EST or mRNA sequences representing transcribed pseudogenes

A total of 10 ABC transporter pseudogenes (45%) were intronless, directly flanked by repetitive sequences (as identified with the RepeatMasker track, UCSC Genome Browser), and most of them (n = 8) also exhibited poly(A)-tails. These structural features are diagnostic of processed pseudogenes [10] strongly suggesting that they originated from retrotransposed transcripts of ancestral functional ABC transporter genes (Table 1).

### A significant portion of human ABC transporter pseudogenes are transcriptionally active

**Figure 2:**
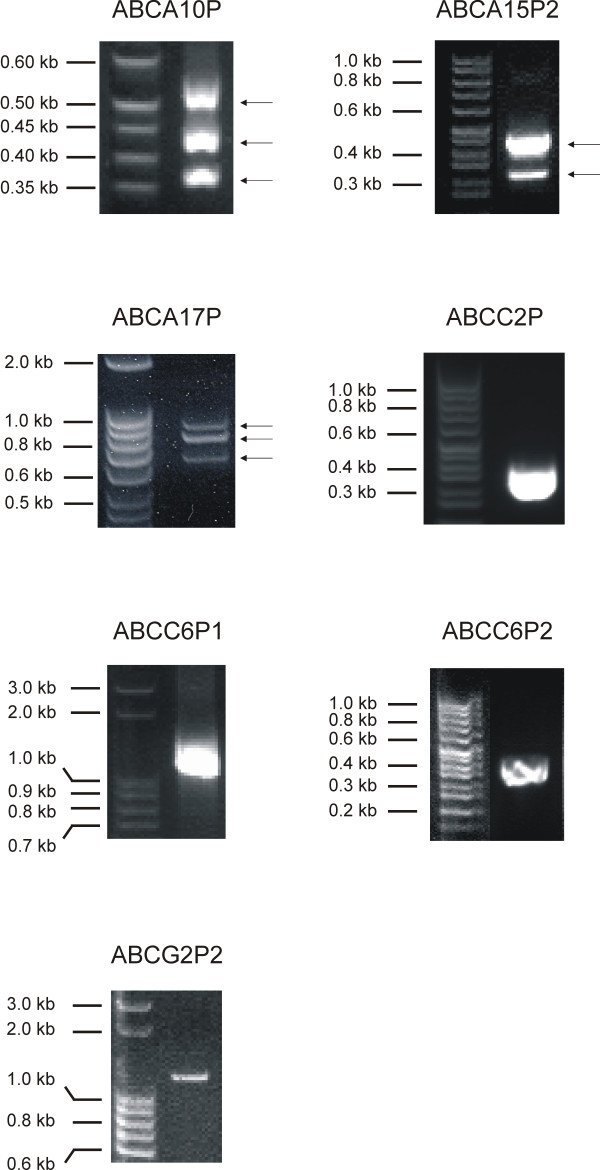
**Expression of human ABC transporter pseudogene RNAs**. RT-PCR expression profiling demonstrating that the ABC transporter pseudogenes *ABCA10P*, *ABCA15P2*, *ABCA17P*, *ABCC2P*, *ABCC6P1*, *ABCC6P2 *and *ABCG2P2*, respectively, are transcribed as predicted by our *in silico *results.RT-PCR was performed using human cDNA from pooled tissues. Note that *ABCA10P*, *ABCA15P2*, and *ABCA17P *are expressed as alternatively spliced transcript variants (as confirmed by sequence analysis) (arrows).

To assess which of the identified human ABC transporter pseudogenes are transcribed, we analyzed all 22 pseudogenic ABC transporter loci for homologies with EST or mRNA sequences using the UCSC Genome Browser. Using this approach 101 EST and/or mRNA sequences were identified that exhibited best hits (> 99% overall identity) with 10 of the 22 human ABC transporter pseudogenes. These sequences displayed higher sequence identity to their corresponding pseudogene locus than to any other location in the human genome. This indicates that a significant portion (45%) of the human ABC transporter pseudogenes is transcriptionally active (Table 1). Among these, n = 5 (50%) are processed pseudogenes. Consistent with our *in-silico *results, RT-PCR expression analysis of all ABC transporter pseudogene transcripts that span at least two exons demonstrated that the pseudogenes *ABCA10P*, *ABCA15P2*, *ABCA17P*, *ABCC2P*, *ABCC6P1*, *ABCC6P2 *and *ABCG2P2 *are in fact transcribed (Figure 2). Interestingly, we identified alternatively spliced transcripts in three out of the 10 transcribed pseudogenes (*ABCA10P*, *ABCA15P2*, *ABCA17P*).

### Gene structures and expression profiling of *ABCC6 *and its pseudogenes *ABCC6P1 *and *ABCC6P2 *in various human tissues

**Figure 3:**
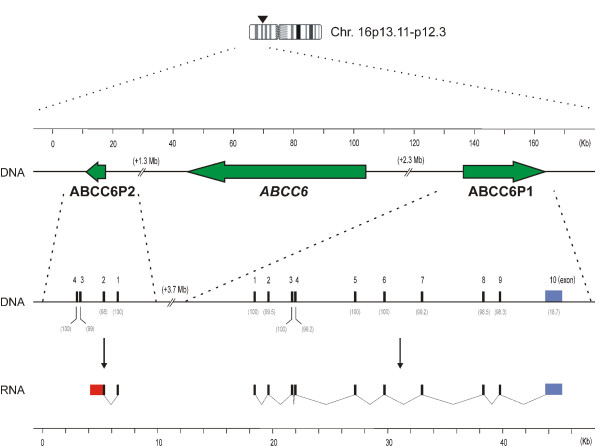
**Genomic organization of *ABCC6P1 *and *ABCC6P2*, two transcribed pseudogenes of *ABCC6***. The pseudogenes *ABCC6P1 *and *ABCC6P2 *flank their parental gene *ABCC6 *at a distance of 2.3 Mb and 1.3 Mb, respectively, on chromosome 16p13. Arrows indicate the direction of transcription. The structures of the *ABCC6P1 *and *ABCC6P2 *genes and their transcripts are illustrated in higher magnification. Exons are represented by black boxes and numbered in 5' to 3' order. Similarities (%) of pseudogene exons with their corresponding *ABCC6 *exons are shown in parenthesis. The terminal non-homologous exon of *ABCC6P1 *is shaded blue. The red box highlights the last exon of *ABCC6P2 *which contains a part of intron 2. Metric scale bars are indicated for orientation.

Two pseudogenes, *ABCC6P1 *and *ABCC6P2*, have been reported for the pseudoxanthoma elasticum gene *ABCC6*, a member of the ABC C-subfamily [7]. They are located 2.3 Mb centromeric (*ABCC6P1*) and 1.3 Mb (*ABCC6P2*) telomeric, respectively, of the protein coding *ABCC6 *gene (Figure 3). When we performed a detailed bioinformatic analysis of both pseudogenes, we retrieved a poly(A)-containing 2.7 kb mRNA from the GenBank NCBI database which had been isolated from human liver cells during the course of the Mammalian Gene Collection Program [11]. The transcript [GenBank: BC075833] exhibits perfect homology with the 9 putative exons of *ABCC6P1 *indicating that this pseudogene is transcriptionally active. Comparison of the genomic sequence and the 2.7 kb transcript revealed that the *ABCC6P1 *gene consists of 10 exons (Figure 3). The *ABCC6P1 *intron sizes range from 0.1 to 5.2 kb. Exons 1–9 have perfect or near perfect identity (98.3%–100%) to the corresponding exons of the parental gene *ABCC6*. In contrast, the final exon (exon 10) of *ABCC6P1 *shows no homology to *ABCC6*. Due to the observed sequence disparity in exon 10, the transcripts of *ABCC6P1 *and *ABCC6 *can be reliably distinguished by RT-PCR. Moreover, it allows selective targeting of the pseudogene transcript using RNA interference strategies.

We also found *in silico *evidence for transcription of the second *ABCC6 *pseudogene, *ABCC6P2*. Consistent with this, our RT-PCR experiments using pooled mRNA from 20 human tissues confirmed expression of this pseudogene (Figure 2). *ABCC6P2 *is composed of four exons whose lengths vary between 59–183 bp which exhibit highest sequence similarity (98–100%) with the corresponding exons of its parental gene. Intron sizes of *ABCC6P2 *range from 0.1 to 1.7 kb. Interestingly, Genbank mRNA database searches revealed a 0.7 kb poly(A)-tail transcript which displays perfect identity with *ABCC6P2 *exons 1 and 2 plus additional 504 bp of the second intron (Figure 3). This transcript has been independently identified in the Mammalian Gene Collection Program [11] from the kidney [GenBank: BC015978].

**Figure 4:**
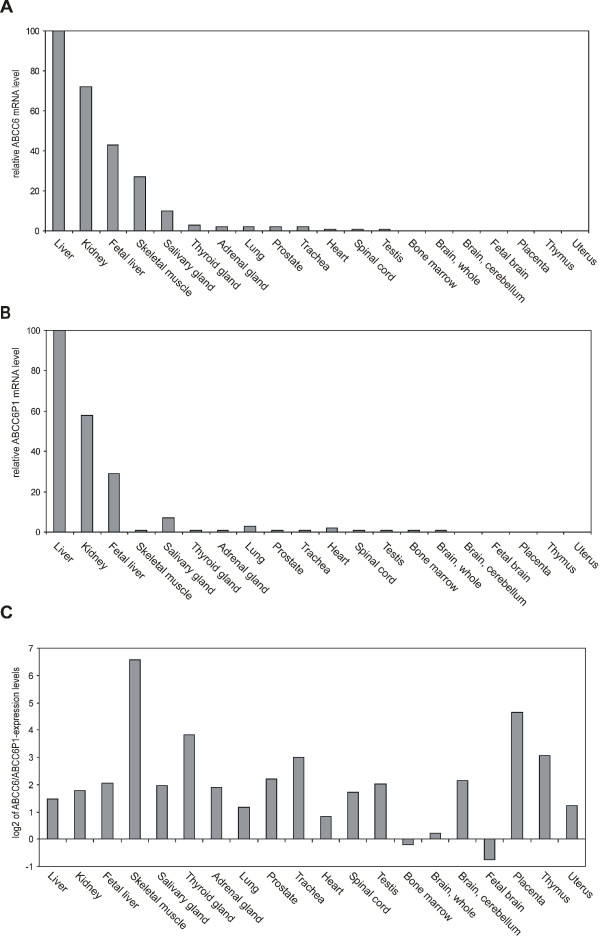
***ABCC6 *and its transcribed pseudogene *ABCC6P1 *are co-expressed in a variety of human tissues**. Relative transcription levels of *ABCC6 ***(A) **and *ABCC6P1 ***(B) **were determined in 20 different human tissues by real-time RT-PCR relative to those of the housekeeping gene β-actin. Highest expression of both *ABCC6 *and *ABCC6P1 *was observed in adult liver (reference = 100%), kidney and fetal liver. **(C) ***ABCC6*/*ABCC6P1 *transcript ratios. Note that *ABCC6 *and *ABCC6P1 *are co-expressed in all tissues investigated with the *ABCC6 *expression levels being on average 2–4 times higher than those of *ABCC6P1*. Ratios are shown in a logarithmic scale (base 2).

We next tested whether and to which degree regulatory interdependencies exist between *ABCC6 *and *ABCC6P1*. For this, we performed expression profiling of both genes in various human tissues by real-time PCR. Expression levels were normalized to the expression of the housekeeping gene β-actin. We observed a similar gene expression pattern for *ABCC6 *and *ABCC6P1 *with highest expression levels in liver and kidney (Figure 4A, B). In most tissues *ABCC6 *mRNA levels were moderately higher (on average 2–4 fold) than those of *ABCC6P1*, however, *ABCC6P1 *was the predominant transcript in bone marrow and fetal brain (Figure 4C). Similar results were obtained when the expression levels were normalized to cyclophilin B, another housekeeping gene (data not shown).

The striking co-expression of the *ABCC6 *and *ABCC6P1 *raises the possibility that transcription of both genes is controlled by similar regulatory elements. When we compared the 5' regulatory region sequences of both genes, we found that they share an overall sequence similarity of 98.9% within the first 2.5 kb upstream of the start codon, the region that contains the putative core promoters (Additional File 2). Of note, 68 out of the 72 potential transcription factor binding sites, which we identified within the predicted *ABCC6P1 *promoter region, are fully intact. In particular, an NF-κB consensus binding sequence (pos. -233 bp), known to confer high-level *ABCC6 *expression in HepG2 hepatoma cells [12], is completely conserved in the putative *ABCC6P1 *promoter region. Three binding sites, a glucocorticoid response element (GR, pos. -2116 bp), an octamer binding factor (oct-1, pos. -1746 bp) and a GATA-3 consensus site (pos. -1269 bp), respectively, show single base exchanges. In addition, a USF binding site (pos. -622 bp) exhibits a nonamer-deletion which disrupts the respective consensus sequence. Intriguingly, single base exchanges give rise to five novel transcription factor binding sites (SRY -2381 bp, C/EBPb -1223 bp, GATA-2 -1124 bp, AML-2 -464 bp, C/EBP -316 bp) in the predicted *ABCC6P1 *promoter.

Comparison of the *ABCC6 *promoter sequence with the *ABCC6P2 *5' UTR revealed an even higher degree of homology (99.6%) than that observed for *ABCC6P1*. A total of 71 out of 72 transcription factor binding sites were fully conserved strongly suggesting that the *ABCC6P2 *expression pattern is highly similar to that of *ABCC6 *and *ABCC6P1*, respectively. We were unable, however, to quantitate *ABCC6P2 *expression levels by real-time PCR because the small size of the transcript and its exceedingly high sequence homology with *ABCC6 *and *ABCC6P1 *hampered specific detection of *ABCC6P2 *mRNA.

### Targeted knockdown of the pseudogene *ABCC6P1 *results in downregulation of ABCC6 mRNA expression

**Figure 5:**
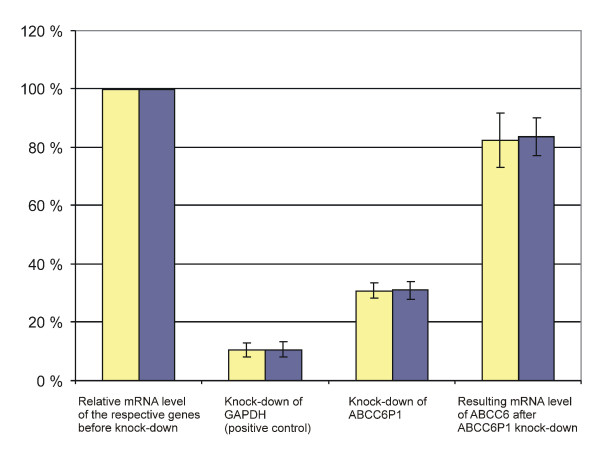
**Silencing of the transcribed pseudogene *ABCC6P1 *downregulates mRNA levels of *ABCC6***. Targeted siRNA-mediated inhibition of *ABCC6P1 *expression in HepG2 cells results in significant downregulation (69%, 95%-CI: 65–73%) of the pseudogene transcript levels (lane 3). mRNA levels are normalized to β-actin (yellow bars) and cyclophilin B (blue bars). *ABCC6P1 *silencing is associated with a reduction of *ABCC6 *mRNA expression (84%, p = 0.003, 95%-CI: 76–91%, yellow bar; 82%, p = 0.01, 95%-CI: 72–93%, blue bar, lane 4). A positive control (knock-down of *GAPDH*) is shown (lane 2). Data were obtained from six independent experiments. The relative expression levels before knock-down experiments were set to 100% (lane 1).

The *ABCC6*/*ABCC6P1 *gene-pseudogene pair is unique in that (i) both components exhibit similar expression patterns, (ii) share the extensive similarity of their (putative) promoter regions and (iii) are positioned in close spatial proximity on chr. 16p12. The combination of these features raises the question as to whether regulatory interdependencies exists between *ABCC6P1 *and its paralog *ABCC6*. To address this question we tested whether silencing of *ABCC6P1 *has an effect on *ABCC6 *expression. For this, a siRNA-mediated gene knockdown approach targeting the non-homologous exon 10 of *ABCC6P1 *was used which allows selective silencing of *ABCC6P1 *but not *ABCC6*. Among several exon10 specific siRNAs, we selected the one by which the most efficient gene silencing could be obtained in HepG2 liver cells. Knockdown effects were normalized to the housekeeping genes β-actin and cyclophilin B, respectively. Using this approach, an average 69% (SD 4%, 95%-CI: 65–73%) knockdown of the *ABCC6P1 *pseudogene was achieved in six independent experiments (Figure 5, lane 3). Importantly, knockdown of *ABCC6P1 *led to a significant reduction of *ABCC6 *mRNA expression. The residual expression of *ABCC6 *was 84% (SD 7%, p = 0.003, 95%-CI: 76–91%), when normalized to β-actin, and 82% (SD 11%, p = 0.01, 95%-CI: 72–93%) relative to cyclophilin B, respectively (Figure 5, lane 4). No significant changes in *ABCC6 *or *ABCC6P1 *expression levels were observed in HepG2 cells when unspecific siRNA were used or lipid transfection agent alone were used (negative controls, data not shown). In contrast, *GAPDH*-specific siRNA (positive control) showed an 89% (95%-CI: 87–97%) knockdown relative to β-actin and cyclophilin B (Figure 5, lane 2). Similarity searches using the *ABCC6P1 *siRNA target sequence (21 bp) as a bait consistently showed mismatches > 5 bp throughout the entire promoter region and all exons and introns of *ABCC6*. Therefore, the siRNA mediated suppression of *ABCC6 *cannot be attributed to "co-targeting" of a common sequence motif in both genes. Our finding that silencing of *ABCC6P1 *has a suppressive effect on *ABCC6 *thus suggest that the expression of *ABCC6P1 *and its parental gene are interdependent.

## Discussion

In our study, we report the existence of 22 human ABC transporter pseudogenes. The total number of pseudogenes in the human genome has been estimated to ~20000 [13-15], whereas the predicted number of protein coding genes amounts to 20000 – 25000 [16]. This estimation indicates a genome-wide gene/pseudogene ratio of app. 1:1. Given the existence of 48 functional human ABC transporters, the ABC transporter genes/pseudogene ratio is app. 2:1. Thus, the number of ABC transporter pseudogenes in the human genome is significantly lower than the expected average frequency of pseudogenes within currently identified gene-pseudogene families [17-19].

Our results indicate that the pseudogenes of the ABC transporter family are distributed unevenly across the chromosomes. Approximately one-half of the ABC transporter pseudogenes (n = 11) is dispersed across several chromosomes, whereas the other half is clustered on chr. 15 and 16, respectively. Of these, seven pseudogenes are located on the short arm of chr. 16 within a segment of 30 Mb (16p11.2-p13.3) and three processed (intronless) pseudogenes are clustered on the long arm of chr. 15. The ABC transporter pseudogene cluster on chr. 16p consists of three protein-coding ABC transporter genes, six duplicated pseudogenes and one processed pseudogene. Clustering of pseudogenes has been observed for other gene families in various vertebrates including the group of cytochrome P450 enzymes, α-globin, and the variable gene segments of the immunoglobulin heavy and light genes, respectively [17,20,21]. These gene clusters are usually composed of both protein coding genes and pseudogenes from the same gene family, which likely reflects the fact that pseudogenization is a mechanism that is highly associated with gene duplication events. The close spatial proximity of genes and pseudogenes suggests that the gene duplications are more recent evolutionary events because dispersion across the genome has not yet occurred. In light of this, it is reasonable to assume that the ABC transporter pseudogenes located on chr. 15 and 16 are indeed the result of gene duplication and subsequent pseudogenization of the previously functional ABC transporter genes. Of note, the rodent genome contains protein-coding genes for *Abca14*, *Abca15 *[22] and *Abca17 *[23]. It is thus conceivable that the human orthologs of these intact genes were functional for a certain time period after duplication before they ultimately became disabled. As for ABCC6P1 and ABCC6P2, the other two duplicated pseudogenes on chr 16, it remains unclear whether they arose by complete duplication of the parental ABCC6 gene and thus may have temporarily maintained their function or whether only fragments of this gene were duplicated *ab ovo*.

Unlike in the case of non-processed pseudogenes, the chromosomal distribution of processed pseudogenes generally appears to be random with the number of pseudogenes per chromosome being proportional to chromosomal length [15]. This is consistent with the view that processed pseudogenes originate from nondirected retrotransposition of preexisting transcripts. Interestingly, the pseudogene cluster on chr. 15 contains three processed pseudogenes (*ABCB10P1*-*3*) which are localized in close proximity to each other (their parental gene *ABCB10 *is located on chr. 1). Because it is unlikely that the *ABCB10P *cluster is the result of three independent retrotransposition events targeting one and the same chromosomal segment, two of the three *ABCB10 *pseudogenes most likely arose by gene duplication. The homology analyses, which we conducted, indeed suggest that *ABCB10P1 *may have originated from *ABCB10 *by a retrotransposition process. Intriguingly, they also strongly support the view that *ABCB10P2 *arose by duplication of *ABCB10P1 *and *ABCB10P3 *is the result of the duplication of *ABCB10P2 *(Additional File 3). Because *ABCB10P2 *and *ABCB10P3 *most likely arose by duplication of an already disabled processed pseudogene, it is reasonable to assume that these pseudogenes never had the capacity to encode proteins. To our knowledge, the *ABCB10P *cluster thus represents the first example of the multiplication of a processed pseudogene in the human genome.

Importantly, we obtained bioinformatic evidence indicating that app. one-half (45%) of the human ABC transporter pseudogenes are transcriptionally active. Consistent with this, our RT-PCR based survey demonstrated that at least 7 out of the predicted transcriptionally active pseudogenes were in fact transcribed. Little attention has so far been paid to the phenomenon of pseudogene transcription [6] owing to the fact that pseudogenes are generally thought to be functionless genomic relics. However, this view is challenged by some recent studies. For example, a comprehensive study focussing on transcription of processed pseudogenes in the human genome revealed that a subfraction of app. 5% of all processed pseudogenes identified are transcribed [24]. Moreover, analysis of pseudogene transcription on human chr. 22 using a combined bioinformatic/microarray approach demonstrated that up to 20% of the pseudogenes identified on this chromosome are transcriptionally active [25]. The surprisingly high proportion of potentially transcribed pseudogenes questions the traditional view of pseudogenes and raises the intriguing possibility that transcribed pseudogenes may serve defined biological functions in the genome. The concept is supported by two recent studies which provide experimental evidence for pseudogene function. Korneev et al. reported direct interference of a gene and its transcribed pseudogene by demonstrating that the antisense pseudogene *anti-NOS*, whose transcript forms stable RNA-RNA complexes with the mRNA of *nNOS*, inhibits protein expression of its parental gene [26]. Another study highlights the fact that pseudogene expression can exert a biological function that is unrelated to its ancestral gene as evidenced for the human *Xist *noncoding RNA gene, which initiates X chromosome inactivation and has evolved in eutherians by pseudogenization of the protein coding gene *Lnx3 *[27].

To test whether and to which degree regulatory interdependencies exist between transcribed ABC transporter pseudogenes and their protein coding counterparts, we selected the pseudoxanthoma elasticum gene, *ABCC6*, and its transcribed pseudogene *ABCC6P1*. This gene-pseudogene pair appeared to be an ideal candidate for interference studies because *ABCC6 *and *ABCC6P1 *exhibit near perfect sequence homology in their transcripts but, at the same time, can still be discriminated by RT-PCR due to non-homology disparity in the final exon (exon 10) of *ABCC6P1*. Moreover, this sequence disparity allowed the selective targeting of the pseudogene transcript using an siRNA approach. mRNA expression profiling of *ABCC6 *and *ABCC6P1 *revealed that both genes are consistently co-expressed in a wide range of human tissues with predominant expression in adult liver, kidney and fetal liver (in this order). The tissue expression pattern we observed for ABCC6 is in keeping with a recent genome-wide gene expression study involving 79 human tissues [28]. Regrettably, this work does not include expression data on the pseudogene ABCC6P1. Our finding that *ABCC6 *and *ABCC6P1 *share near-perfect identity in their respective core promoter regions together with the observed co-expression pattern strongly suggests that the expression of the gene-pseudogene pair is governed by the same transcription factor machinery.

Intriguingly, we found that selective knockdown of *ABCC6P1 *leads to a reduction of *ABCC6 *mRNA expression suggesting that the expression of *ABCC6P1 *and its parental gene are interdependent. At this point, the regulatory mechanisms which underlie the observed gene-pseudogene interference remain speculative. It is well established that mRNA stability and degradation significantly depends on specific *cis*-acting elements in mRNA molecules. These cis-acting destabilizing elements, which can be found both in the UTR and the coding regions of mRNA are attacked by *trans*-acting machineries facilitating mRNA decay [29]. Transcripts from distinct genes which exhibit extensive sequence homology (as in the case of gene-pseudogene pairs) and thus contain identical destabilizing cis-elements may compete for such *trans*-acting elements. It is thus conceivable that the *ABCC6 *and the *ABCC6P1 *transcripts due to their high sequence similarity and their topological proximity compete for the same cellular RNA degradation route. As a consequence, lower abundance in *ABCC6P1 *transcripts may thus lead to an increased *ABCC6 *mRNA degradation rate. It will be challenging to characterize in more detail the molecular interdependency between the *ABCC6 *and *ABCC6P1 *transcription units. Knowledge of *ABCC6*/*ABCC6P1 *regulatory interaction may be of potential relevance to clinical medicine because *ABCC6 *is the underlying gene defect in the syndrome pseudoxanthoma elasticum.

## Conclusion

In this study, we established the existence of 22 ABC transporter pseudogenes in the human genome. Combined bioinformatic and experimental results indicate that a significant portion of these pseudogenes (45%) are transcribed and we provide evidence, for the first time, for expression of alternatively spliced pseudogene RNAs in the human genome. Moreover, we show that *ABCC6P1*, one of the pseudogenes of the pseudoxanthoma elasticum gene *ABCC6*, is co-expressed with ABCC6 in a variety of tissues and that knockdown of *ABCC6P1 *expression impacts the expression of *ABCC6 *strongly suggesting a regulatory interdependence between *ABCC6P1 *and its ancestral gene. The emerging concept of pseudogene transcription and functionality has recently found its reflection in the redefinition of pseudogenes as "genomic sequences that arise from functional genes but that cannot encode the same type of functional product [...] as the original gene" [30]. Accordingly, a subclassification into (i) 'ghost' pseudogenes with some intermediate functionality, i.e. similar to a functional gene and transcribed and/or functional, and (ii) 'dead' pseudogenes that lack any functionality has recently been proposed. Our results presented here strongly support this novel differentiated view on the biology of pseudogenes.

## Methods

### Bioinformatic identification of human ABC transporter pseudogenes

To identify all ABC transporter pseudogenes in the human genome, we retrieved the complete sequence of the human genome [31] chromosome-wise from the UCSC Genome Browser (assembly March 2006, NCBI Build 36, hg18) [32,33].

A stand-alone version of the program MaskSeq was used to mask off the exons of all human reference ABC transporter genes. For this, a list of the reference nucleotide sequence accession numbers of all human ABC transporters was used to retrieve the exon coordinates of the human protein coding ABC transporter genes from the Table Browser application of the UCSC Genome Browser [34]. A Perl script was designed to extract and reformat the exon coordinates from the Table browser output for application in the program MaskSeq.

In addition to all human reference ABC transporter sequences, we downloaded the reference nucleotide sequences of all known mouse ABC transporters that have no apparent orthologous counterparts in the human genome (*Abca14*, *Abca15*, *Abca16*, *Abca17*, *Abcg3*). Using a local installation of the BLAST application (version 2.2.14), a homology search of the reference ABC transporter nucleotide sequences was performed against the human genome masked for ABC transporter exons. In this alignment, an E-value of 0.001 and the table output format "-m8" was applied. To eliminate hits originating from repetitive sequences, the repetitive sequences in the query ABC transporter nucleotide sequences were identified by applying the program RepeatMasker (version 3.1.6) using the database Repbase (version 11.12). An in-house written Perl script was used to exclude all hits that were located completely within repetitive sequences and those that extended less than 30 bp into the flanking regions of repetitive sequences. The remaining hits were then ordered along the chromosomes and merged to a single candidate pseudogene when neighbouring hits yielded highest homology to the same protein coding gene and when they were in the same 5'→3' orientation as their homologous ABC transporter nucleotide sequence. In the absence of a neighbouring hit, a genomic region of 0.1 Mb flanking the single hit was aligned with the respective ABC transporter nucleotide sequence using the more rigorous alignment tool "matcher" from the EMBOSS package. This approach allows for identification of more distantly related pseudogenes. Hits displaying > 70% identity over an entire exon of the respective ABC transporter nucleotide sequence, were also considered homologous and further evaluated. Merged hits were classified as candidate pseudogenes when (i) the retrieved sequences showed homologies to at least two exons of a protein coding gene and (ii) the merged hit contained sequence aberrations precluding its translation into a functional ABC transporter. Subsequently, all potential pseudogenes were analyzed using the UCSC Genome Browser [33] to exclude the possibility that candidate pseudogenes were part of a known protein coding gene. Additionally, the human mRNA and EST track of the UCSC Genome Browser were used to search for transcription of the identified ABC transporter pseudogenes. A locus was regarded a candidate transcriptionally active pseudogene when a homologous mRNA or EST sequence exhibited overall > 99% with the pseudogene but no other sites in the human genome ("best hit").

Sequence similarities and multiple sequence alignments were calculated using EMBOSS package algorithms [35,36]. Potential transcription factor binding sites were identified utilizing tfsearch (version 1.3, threshold score 85.0, classification: vertebrate) [37] and the TRANSFAC database (release 3.3) [38].

### Confirmation of pseudogene transcription

Aliquots of 1 μg pooled total RNA (Human Total RNA Master Panel II, Clontech) were reverse-transcribed in the presence of oligo-dT primers in a 20 μl RT reaction using the Omniscript RT Kit (Qiagen). cDNA was amplified using the HotStarTaq DNA polymerase Kit (Qiagen). The cycling conditions were 94°C 30s, 54–60°C 30s and 72°C 30s-3 min followed by a final extension step at 72°C for 10 min. Transcription of pseudogenes was authenticated utilizing the following primer sets: *ABCA10P *5'-GATTTTGCCAGAGCCTCAGC-3', 5'-GAGTGCATTTGCTGCTTGGA-3', *ABCA15P2 *5'-AACTGGCATGGACCCAGTAG-3', 5'-CTTCCTCCAAAATGCCAAAC-3', *ABCA17P *5'-TCACGCACTGTCTTTCCTTG-3', 5'-CCAGTACAATGGAACTGATGATG-3', *ABCC2P *5'-GCCTGGATTCAGAATTGCAT-3', 5'-GCCCCACAAGAAAGCATAAA-3', *ABCC6P1 *5'-CCCACGACGACAGAAGG-3', 5'-TTCGGAAACCTTGGCTCA-3', *ABCC6P2 *5'-CCCACGACGACAGAAGG-3', 5'-TCTGTTTGGGAGAACCGTGT-3' and *ABCG2P2 *5'-GGTACTCCGCTGACACCTTC-3', 5'-TTTCTCAACTGGTTTTTGACCA-3'. PCR products were separated and analyzed on GelStar^® ^(Cambrex) stained 0.8 – 1.5% agarose gels. Bands of interest were excised and the DNA was extracted using the QIAquick^® ^Gel Extraction Kit (Qiagen). PCR runs resulting in a single band were purified using Microcon^® ^YM-30 columns (Millipore) for subsequent sequence analysis. Purified amplification products were sequenced as published previously [39]. Sequence data were analyzed using the program FinchTV v.1.3.1 (Geospiza Inc.) and the UCSC Genome Browser.

### Cell culture

HepG2 cells were purchased from ATCC (no. HB-8065) and cultivated under standard conditions (5% CO2, 37°C) in ATCC complete growth medium (Eagle's medium) with 2 mM L-glutamine and Earle's BSS (1.5 g/L sodium bicarbonate, 0.1 mM non-essential amino acids, 1.0 mM sodium pyruvate, 90%; fetal bovine serum, 10%) containing 2% penicillin/streptomycin.

### siRNA mediated knockdown of ABCC6P1 in HepG2 cells

21 mer-siRNAs were designed using the siRNA design tool from the Whitehead Institute for Biomedical Research [40], which target the sequence of exon 10 of the pseudogene *ABCC6P1*. This exon displays no homology (sequence identity < 20%) with its parental gene *ABCC6 *and thus allows selective targeting of *ABCC6P1*. siRNAs including positive (Silencer *GAPDH *siRNA) and negative controls (Silencer Negative Control #1 siRNA) were purchased from Ambion, Inc. After screening of several siRNAs, the following siRNAs which yielded the highest knockdown rates of *ABCC6P1 *were used in all further experiments: Sense 5'-CUCCCAGGGAUAUAAAUCAUU-3', and antisense 5'- UGAUUUAUAUCCCUGGGAGUU-3', respectively. HepG2 cells were reverse-transfected using 1.5 μl siRNA (20 μM) complexed with 3 μl siPORT™ NeoFX™ transfection agent (Ambion) in 100 μl OPTI-MEM buffer (Invitrogen). After 10 min incubation, the siRNA/lipid-complex was mixed with 900 μl of 10^5^cells/ml HepG2 cells in culture medium and plated in 12 well plates. After 24 h incubation under standard conditions the cells were lysed, RNA was extracted (MagNA Pure LC RNA Isolation Kit – High Performance, Roche) and quantitated on a Nano Drop^® ^spectrophotometer. All siRNA experiments were performed in duplicate, and the results of six independent siRNA experiments with *ABCC6P1 *knockdown rates > 60% were used for statistical analyses.

### Quantitative RT-PCR

For gene expression profiling of *ABCC6 *and *ABCC6P1 *in different human tissues, 1 μg total RNA from each tissue type of Clontech's Human Total RNA Master Panel II, and for quantitation of transcription levels in the siRNA experiments, 150–250 ng total RNA from HepG2 cells were reverse transcribed using Omniscript (Qiagen) and oligo-dT primers. For quantitation of the transcription levels of selected genes real-time PCR was performed on a 7900HT Fast Real-Time PCR System (Applied Biosystems) using standard conditions. The following TaqMan-primer/probes (Applied Biosystems) were used: *ABCC6P1 *(designed by Applied Biosystems): forward primer 5'-CCATCACTGGCCTGGTGTA-3', reverse primer 5'-CTCCTGCTGAAATCATCTGAGATTAGT-3', FAM-reporter probe 5'-CTGTGGATGCCTTTCTG-3' (targeting exon boundary 9/10 of *ABCC6P1*), *ABCC6 *(Assay-ID: Hs01081199_m1, targeting exon boundary 25/26 of *ABCC6*), cyclophilin B (*PPIB*) (Assay-ID: Hs00168719_m1), β-actin *(ACTB) *(Assay-ID: Hs99999903_m1), *GAPDH *(Assay-ID: Hs99999905_m1). All RT-PCR experiments were performed at least in duplicate.

### Statistic analyses and calculation of gene/pseudogene expression levels

Relative transcript levels were calculated using the ΔΔ-ct method for the two housekeeping genes β-actin and cyclophilin B. Means, p-values and confidence intervals were calculated using the software package SPSS.

## Authors' contributions

APP, MH, PK, WEK designed the study. EJ, KBFH, PK, WEK supervised the project. APP, JJW performed bioinformatic analyses. APP, MH performed cell culture, siRNA and gene expression experiments. APP, MH, JJW, KBFH, WEK analyzed the data. APP, MH, EJ, WEK prepared the manuscript. All authors read and approved of the final manuscript.

## Supplementary Material

Additional file 1 Repetitive elements in ABC transporter reference nucleotide sequences. Click here for file

Additional file 2 **Comparison of the 5'UTR regions of *ABCC6*, *ABCC6P1 *and *ABCC6P2***. The overall homology between the 5' upstream regions of *ABCC6 *and those of *ABCC6P1 *and *ABCC6P2 *is 98.8% and 99.5%, respectively. Transcription factor consensus binding sites are shown in green black. Importanty, all potential transcription factor binding sites within the *ABCC6 *promoter, with few exceptions (glucocorticoid response element GR -2116 bp, octamer binding factor oct-1 -1746 bp, GATA-3 consensus site -1269 bp and USF binding site -622 bp), are also present in *ABCC6P1 *and *ABCC6P2*. Non-homologous nucleotides are black white, the transcription start site of *ABCC6 *is indicated (+1). Click here for file

Additional file 3 Similarities (%) between the members of the *ABCB10 *gene-pseudogene cluster. Click here for file
