# Supplementary material for: The human ABC transporter pseudogene family: Evidence for transcription and gene-pseudogene interference
Source: BMC Genomics. 2008 Apr 11;9:165. doi: 10.1186/1471-2164-9-165 (PMC2329642; doi:10.1186/1471-2164-9-165)
Supplement: Additional file 1 — Repetitive elements in ABC transporter reference nucleotide sequences. [file 1471-2164-9-165-S1.pdf]

Additional File 1. Repetitive elements in ABC transporter reference nucleotide sequences.

ABC-ID	Position in cDNA Start	End	Matching repeat	Repeat family
**ABCA**				
ABCA1	7234	7258	(CAATG)n	Simple repeat
	8549	8569	AT_rich	Low complexity
ABCA2	52	142	(CGCGG)n	Simple repeat
	8131	8172	AT_rich	Low complexity
ABCA3	6018	6106	CR1_Mam	LINE/CR1
	6468	6491	(A)n	Simple repeat
ABCA5	1	131	AluSg	SINE/Alu
	175	1129	L1MD2	LINE/L1
ABCA8	3595	3632	CT-rich	Low complexity
	5231	5260	AT_rich	Low complexity
ABCA9	5351	5400	AT_rich	Low complexity
	5589	6079	L1PA7	LINE/L1
ABCA13	15988	16105	MER5A	DNA/MER1_type
	16201	16508	AluY	SINE/Alu
	16513	16537	AT_rich	Low complexity
	17186	17208	(A)n	Simple repeat
**ABCB**				
TAP1 (ABCB2)	2952	2974	(A)n	Simple repeat
TAP2 (ABCB3)	2391	2412	(GGGTG)n	Simple repeat
	2529	2659	L1ME4a	LINE/L1
	2822	3041	MIRb	SINE/MIR
	3360	3761	L2c	LINE/L2
	5111	5316	MIRb	SINE/MIR
	5294	5374	L2	LINE/L2
	5375	5400	AT_rich	Low complexity
	5633	5657	AT_rich	Low complexity
ABCB4	3930	3957	AT_rich	Low complexity
ABCB6	2973	2993	(A)n	Simple repeat
ABCB7	2383	2404	AT_rich	Low complexity
ABCB10	441	470	GC_rich	Low complexity
**ABCC**				
ABCC1	36	78	(CCG)n	Simple repeat
	109	190	GC_rich	Low complexity
ABCC2	4722	4861	L1ME3B	LINE/L1
ABCC3	4814	4937	MIR	SINE/MIR
ABCC4	86	112	GC_rich	Low complexity
	457	484	AT_rich	Low complexity
	5833	5871	(A)n	Simple repeat
CFTR (ABCC7)	5774	5802	AT_rich	Low complexity
ABCC8	1	44	GC_rich	Low complexity
	3026	3074	GA-rich	Low complexity
ABCC9	2870	2914	GA-rich	Low complexity
ABCC10	1	41	L1PA17	LINE/L1
	5089	5118	(A)n	Simple repeat
ABCC12	4952	4979	AT_rich	Low complexity
**ABCD**				
ABCD1	41	76	GA-rich	Low complexity
ABCD2	3645	3863	MIRc	SINE/MIR
	3949	3969	(A)n	Simple repeat
ABCD3	3373	3392	(A)n	Simple repeat
ABCD4	2758	2881	MER113	DNA/MER1_type
	2888	2927	(A)n	Simple repeat
ABCE1	3061	3152	MER5B	DNA/MER1_type
**ABCF**				
ABCF1	283	313	(CAG)n	Simple repeat
	779	823	GA-rich	Low complexity
ABCF2	2712	2894	MER5A	DNA/MER1_type
	2974	3227	AluJb	SINE/Alu
	3236	3534	AluJo	SINE/Alu
ABCF3	39	62	GC_rich	Low complexity
**ABCG**				
ABCG1	3474	3498	(A)n	Simple repeat
ABCG2	2755	2852	MIR3	SINE/MIR
	3175	3233	MER5A	DNA/MER1_type
	3469	3585	L1ME3A	LINE/L1
	3710	3973	L1MD3	LINE/L1
	4389	4445	AT_rich	Low complexity
ABCG4	155	200	(CCCCG)n	Simple repeat
	292	319	GC_rich	Low complexity
	3835	3874	(A)n	Simple repeat
**Murine ABC**				
Abca15	5225	5256	(TTTC)n	Simple repeat
Abcg3	2831	2880	AT_rich	Low complexity
