# Supplementary material for: The human ABC transporter pseudogene family: Evidence for transcription and gene-pseudogene interference
Source: BMC Genomics. 2008 Apr 11;9:165. doi: 10.1186/1471-2164-9-165 (PMC2329642; doi:10.1186/1471-2164-9-165)
Supplement: Additional file 2 — Comparison of the 5'UTR regions of ABCC6, ABCC6P1 and ABCC6P2. The overall homology between the 5' upstream regions of ABCC6 and those of ABCC6P1 and ABCC6P2 is 98.8% and 99.5%, respectively. Transcription factor consensus binding sites are shown in green black. Importanty, all potential transcription factor binding sites within the ABCC6 promoter, with few exceptions (glucocorticoid response element GR -2116 bp, octamer binding factor oct-1 -1746 bp, GATA-3 consensus site -1269 bp and USF binding site -622 bp), are also present in ABCC6P1 and ABCC6P2. Non-homologous nucleotides are black white, the transcription start site of ABCC6 is indicated (+1). [file 1471-2164-9-165-S2.pdf]

Additional File 2. Alignment of the ABCC6 core promoter region with the corresponding 5' upstream regions of ABCC6P1 and ABCC6P2

```
                                                                   GATA-2,MZF1          SRY
ABCC6     CCAAGATATAGTGAATAATATCACAGGGGGTACACCCACAGTATATTAGGGGTAATAGTATATCCCCAGAATATAACGAATAATATCACAGGGTGTACACCCACCGTGACAATATGGATA  -2339
ABCC6P1   CCAAGATATAGTGAATAATATCACAGGGGGTACACCCACAGTATATTAGGGGTAATAGTATATCCCCAGAATATAACAAATAATATCACAGGGTGTACACCCACCGTGACAATATGGATA
ABCC6P2   CCAAGATATAGTGAATAATATCACAGGGGGTACACCCACAGTATATTAGGGGTAATAGTATATCCCCAGAATATAACGAATAATATCACAGGGTGTACACCCACCGTGACAATATGGATA

                                                                     GATA-2,-3,C/EBP          Oct-1              CRE-BP
ABCC6     ATATCTTCCAGGATGTTACGAATAGTATCAAAGAGTATACACCCACTGTGATATTAGGGGCAATATCTCCACAGAATATTACAAATAATATCAAAAGATGTACACGCACTGTGACATTAG  -2219
ABCC6P1   ATATCTTCCAGGATGTTACGAATAGTATCAAAGAGTATACACCCACTGTGATATTAGGGGCAATATCTCCACAGAATATTACAAATAATATCAAAAGATGTACATGCACTGTGACATTAG
ABCC6P2   ATATCTTCCAGGATGTTACGAATAGTATCAAAGAGTATACACCCACTGTGATATTAGGGGCAATATCTCCACAGAATATTACAAATAATATCAAAAGATGTACACGCACTGTGACATTAG

                Lyf1                                                          GATA-2                       GR        MZF1
ABCC6     GGGTAATATCACCCAAAAATATTACAAATAATATCACAGCATGTACACAATGGTGTACGTTCATTGTGATATTATGATATCCATAGGGTATTACAAATAATAGCACAGGGTGTACCCCCA  -2099
ABCC6P1   GGGTAATATCACCCAAAAATATTACAAATAATATCACAGCATGTACACAATGGTGTACGTTCATTGTGATATTATGATATCCATAGGGTATTACAAATAATATCACAGGGTGTACCCCCA
ABCC6P2   GGGTAATATCACCCAAAAATATTACAAATAATATCACAGCATGTACACAATGGTGTACGTTCATTGTGATATTATGATATCCATAGGGTATTACAAATAATAGCACAGGGTGTACCCCCA

                 GATA-X      Lyf-1                                    GATA-2
ABCC6     CTGTGATATTAGGAGTCATATCTTTCTGGGAGGTCACAGCGTGTACACGCATGGTGTAAATTCACTGGGATATTAGGAGTAATATCGCCCTAGAATATTTCGAATCATATCACAGGGTGT  -1979
ABCC6P1   CTGTGATATTAGGAGTCATATCTTTCTGGGAGGTCACAGCGTGTACACACATGGTGTAAATTCACTGGGATATTAGGAGTAATATCGCCCTAGAATATTTCGAATCATATCACAGGGTGT
ABCC6P2   CTGTGATATTAGGAGTCATATCTTTCTGGGAGGTCACAGCGTGTACACGCATGGTGTAAATTCACTGGGATATTAGGAGTAATATCGCCCTAGAATATTTCGAATCATATCACAGGGTGT

                                                                               GATA-3                               Oct-1
ABCC6     ACACCCACTGTGATATTAAAAGGAATATCTTTCTAGAACATTACAAATAGTATCACAAGGTGTACACCCACTATGAGATTAGGAGTAACATCTCCCTAGAATATTATGAATAATATCACA  -1859
ABCC6P1   ACACCCACTGTGATATTAAAAGGAATATCTTTCTAGAACATTACAAATAGTATCACAAGGTGTACACCCACTATGAGATTAGGAGTAACATCTCCCTAGAATATTATGAATAATATCACA
ABCC6P2   ACACCCACTGTGATATTAAAAGGAATATCTTTCTAGAACATTACAAATAGTATCACAAGGTGTACACCCACTATGAGATTAGGAGTAACATCTCCCTAGAATATTATGAATAATATCACA

                                                                                                                 Oct-1
ABCC6     GTGTGTACAGGCACTGTGATTTTAGGAGTACTACCTTCTTAAGATATTATGAATAATATCATAGGGTATACACCCACTGTGAAATTAAAAGCAATAGCTCCCTACGATATTACGAATAAT  -1739
ABCC6P1   GTGTGTACAGGCACTGTGATTTTAGGAGTACTACCTTCTTAAGATATTATGAATAATATCATAGGGTATACACCCACTGTGAAATTAAAAGCAATAGCTCCCTACGATATTATGAATAAT
ABCC6P2   GTGTGTACAGGCACTGTGATTTTAGGAGTACTACCTTCTTAAGATATTATGAATAATATCATAGGGTATACACCCACTGTGAAATTAAAAGCAATAGCTCCCTACGATATTACGAATAAT

                                       GATA-2                                                     GATA-X
ABCC6     ATCACGCAGTGTACACTCAAGGTGATATTAGGAGTAGTATCTCCCTAGGAAATTACGAATATTATCACAGAGTGTACACCCACTGTGATATTGAAAGTATTATCTTTCTAGGATATTATG  -1619
ABCC6P1   ATCACGCAGTGTACACTCAAGGTGATATTAGGAGTAGTATCTCCCTAGGAAATTACGAATATTATCACAGAGTGTACACCCACTGTGATATTAAAAGTATTATCTTTCTAGGATATTATG
ABCC6P2   ATCACGCAGTGTACACTCTAGGTGATATTAGGAGTAGTATCTCCCTAGGAAATTACGAATATTATCACAGAGTGTACACCCACTGTGATATTAAAAGTATTATCTTTCTAGGATATTATG

                    p300                                                                  AML-1a        GATA-2
ABCC6     AATAATATTACAGGGTGTACTCCCTCTGTGATATTAGGAGTTATATCTCCCTAGGATATTACAAATCATATCACAGGGTGTACACCCACTGTGGTATTAGGTGTAGTATCTCCCTAGGAT  -1499
ABCC6P1   AATAATATTACAGGGTGTACTCCCTCTGTGATATTAGGAGTTATATCTCCCTAGGATATTACAAATCATATCACAGGGTGTACACCCACTGTGGTATTAGGTGTAGTATCTCCCTAGGAT
ABCC6P2   AATAATATTACAGGGTGTACTCCCTCTGTGATATTAGGAGTTATATCTCCCTAGGATATTACAAATCATATCACAGGGTGTACACCCACTGTGGTATTAGGTGTAGTATCTCCCTAGGAT

           SRY
ABCC6     ATAACAAATAATATCACAGGGTATACACCTACGGTTATATCAGGAGTTATATCTTCATAGAATATTATGAATAATATCACAGGGTGTACACCCTCTGTGATATTAAAAGTAATATCTTTC  -1379
ABCC6P1   ATAACAAATAATATCACAGGGTATACACCTACGGTTATATCAGGAGTTATATCTTCATAGAATATTACGAATAATATCACAGGGTGTCCACCCTCTGTGATATTAAAAGTAATATCTTTC
ABCC6P2   ATAACAAATAATATCACAGGGTATACACCTACGGTTATATCAGGAGTTATATCTTCATAGAATATTACGAATAATATCACAGGGTGTACACCCTCTGTGATATTAAAAGTAATATCTTTC

          GATA-2                           Oct-1          Oct-1          GATA-2                                   GATA-3
ABCC6     TAGGATAGTATGAATAATATCACAGGGTGTACTCTCACTGTGATATTAGCGGTAATAACTCCCTAGGATATCACGAATTCTATGACAGAGTGTACACCCACCATGAGATTTGGAGTAATA  -1259
ABCC6P1   TAGGATAGTATGAATAATATCACAGGGTGTACTCTCACTGTGATATTAGCGGTAATAACTCCCTAGGATATCACGAATTCTGTGACAGAGTGTACACCCAC---------TGGAGTAATA
ABCC6P2   TAGGATAGTATGAATAATATCACAGGGTGTACTCTCACTGTGATATTAGCGGTAATAACTCCCTAGGATATCACGAATTCTATGACAGAGTGTACACCCACCATGAGATTTGGAGTAATA

                                        C/EBPb                             GATA-2         Oct-1
ABCC6     TATCTCTAGCATATTATGAATAATATCACAGGTTGTACACCTACTGTGATATTAGGAGTAAAATCTCCCTCGGATATTACAAATAATATCACAGTGTGTACTCCCACTGTGATATTAGGA  -1139
ABCC6P1   TATCTCTAGCATATTATGAATAATATCACAGGTTGCACACCTACTGCGATATTAGGAGTAAAATCTCCCTCGGATATTATAAATAATATCACAGTGTGTACTCCCACTGTGATATTAGGA
ABCC6P2   TATCTCTAGCATATTATGAATAATATCACAGGTTGTACACCTACTGTGATATTAGGAGTAAAATCTCCCTCGGATATTACAAATAATATCACAGTGTGTACTCCCACTGTGATATTAGGA
```

```
         GATA-2   Elk-1, c-Ets   GATA-2   AML-1a   C/EBP
ABCC6    GTAAAATCTCCTTCAGATATTACGAATAATATCACAGGATGTACACCCACTGTGATATTAGGAGTAATATCTCCCTTGGATATTTCTAATAATACCACAGCGTTTACACACATGGTGTTC   -1019
ABCC6P1  GTAAAATCTCCTTCGGATATTATGAATAATATCACAGGATGTACACCCACTGTGATATTAGGAGTAATATCTCCCTTGGATATTTCTAATAATACCACAGCGTTTACACACATGGTGTTC
ABCC6P2  GTAAAATCTCCTTCGGATATTACGAATAATATCACAGGATGTACACCCACTGTGATATTAGAAGTAATATCTCCCTTGGATATTTCTAATAATACCACAGCGTTTACACACATGGTGTTC

         GATA-2   Arnt,USF,c-Myc,Max   GATA-2   GATA-2
ABCC6    ACCCACTGGGATGTTAGGATAATATCTCCCTTGGATATTACCAATCGTATCAGAGGCTGTACACACGTGGTGTTCACCCAGCGTGATATTAGGAATAATATCTCCCTCGGATATTAGGAA   -899
ABCC6P1  ACCCACTGGGATGTTAGGATAATATCTCCCTTGGATATTACCAATCGTATCAGAGGCTGTACACACGTGGTGTTCACCCAGCGTGATATTAGGAATAATATCTCCCTCGGATATTAGGAA
ABCC6P2  ACCCACTGGGATGTTAGGATAATATCTCCCTTGGATATTACCAATCGTATCAGAGGCTGTACACACGTGGTGTTCACCCAGCGTGATATTAGGAATAATATCTCCCTCGGATATTAGGAA

         HSF2   AML-1a   CREB,CRE-BP,GATA-2   Max
ABCC6    CAGTCTCTCCCTGGGACTTAGCAGAACGTAGCCCCCCAGTGTTAAATAAGCGACCACAGCAAGAGGTAGAGGCGCAGGGACTGAAATGAAGACAAATATCTGACAAGTTTAAAACACGTA   -779
ABCC6P1  CAGTCTCTCCCTGGGACTTAGCAGAACGTAGCCCCCCAGTGTTAAATAAGCGACCACAGCAAGAGGTAGAGGCGCAGGGACTGAAATGAAGACAAATATCTGACAAGTTTAAAACACGTA
ABCC6P2  CAGTCTCTCCCTGGGACTTAGCAGAACGTAGCCCCCCAGTGTTAAATAAGCGACCACAGCAAGAGGTAGAGGCGCAGGGACTGAAATGACGACAAATATCTGACAAGTTTAAAACACGTA

         TATA   AML-1a NF-kB   USF,Arnt
ABCC6    TTTAAAATAGAATTTATAAAATGCTTAATCTGCCGACTCAGGAGCCCGCGGTGCAGGGTGGGGTGGGAGTTGGCAGGTGACCGCTACACCAGCAGAGTAAGACTGCAGGGCTGCGGCCCT   -659
ABCC6P1  TTTAAAATAGAATTTATAAAATGCTTAATCTGCCGACTCAGGAGCCCGCGGTGCAGGGTGGGGTGGGAGTTGGCAGGTGACCGCTACACCAGCAGAGTAAGACTGCAGGGCTGCGGCCCT
ABCC6P2  TTTAAAATAGAATTTATAAAATGCTTAATCTGCCGACTCAGGAGCCCGCGGTGCAGGGTGGGGTGGGAGTTGCCAGGTGACCGCTACACCAGCAGAGTAAGACTGCAGGGCTGCGGCCCT

         USF   HSF2   SRY   Sp1
ABCC6    CCCTCCTATGCCCTTCTGTTCAGACACCCGAGGGGCCCATGTGCACTCCTGGGATCATACGACCAGAAAACAGGACCCTAGAGGTTTCTTGAGTTTCTGCTTACCAGGGCGGCTGGGTAT   -539
ABCC6P1  CCCTCCTATGCCCTTCTGTTCAGACACCCGAGGGGCTCATGTGCACTCCTGGGATCATACGACCAGAAAACAGGACCCTAGAGGTTTCTTGAGTTTCTGCTTACCAGGGCGGCTGGGTAT
ABCC6P2  CCCTCCTATGCCCTTCTGTTCAGACACCCGAGGGGCCCATGTGCACTCCTGGGATCATACGACCAGAAAACAGGACCCTAGAGGTTTCTTGAGTTTCTGCTTACCAGGGCGGCTGGGTAT

         C/EBP,HLF,Oct-1   MZF1   AML-2   GATA-2   MZF1
ABCC6    AGCCCTGCCAGCCCATTGCATAATCTTCTAAGTTCTCCCCACCACCCTCCATTCCAGCAGCGAGGTGCCTGTGACGCGGTCTCCATCCTCTCGGCCTCGACCCGGTGGTCCCCGCGATTG   -419
ABCC6P1  AGCCCTGCCAGCCCATTGCATAATCTTCTAAGTTCTCCCCACCACCCTCCATTCCAGCAGCGAGGTGCCTGTGATGCGGTCTCCATCCTCTCGGCCTCGACCCGGTGGTCCCCGCGATTG
ABCC6P2  AGCCCTGCCAGCCCATTGCATAATCTTCTAAGTTCTCCCCACCACCCTCCATTCCAGCAGCGAGGTGCCTGTGACGCGGTCTCCATCCTCTCGGCCTCGACCCGGTGGTCCCCGGGATTG

         C/EBP,Lyf-1
ABCC6    GACGCTTAGGCGGTCACCAGGCCTCCTTGTTACTAACGTGTGCACACCTTTCAGTTCTCTCATCAGGATGAACTCCTGGAAATTGCTGGGTCCAAAGTGTTTAGGAAGTCTGGAGTGATT   -299
ABCC6P1  GACGCTTAGGCGGTCACCAGGCCTCCTTGTTACTAACGTGTGCACACCTTTCAGTTCTCTCATCAGGATGAACTCCTGGAAATTGCTGGGTCCAAAGTGTTTGGGAAGTTTGGAGTGATT
ABCC6P2  GACGCTTAGGCGGTCACCAGGCCTCCTTGTTACTAACGTGTGCACACCTTTCAGTTCTCTCATCAGGATGAACTCCTGGAAATTGCTGGGTCCAAAGTGTTTGGGAAGTTTGGAGTGATT

         MZF1   HSF2   STAT-X,NFkB-like   MZF1,GATA-2,-3
ABCC6    CTTGTTGCAGGGGGAAGAGGGAACTATGGAGGTGTCACTGAACTTTCAGGGGTTCCGGGACCCCCCAACCCGGTGCCCGTCCCAGCTCCCCGAGCGCCTCTCTTCCCCCATCCCCCACCT   -179
ABCC6P1  CTTGTTGCAGGGGGAAGAGGGAACTATGGAGGTGTCACTGAACTTTCAGGGGTTCCGGGACCCCCCAACCCGGTGCCCGTCCCAGCTCCCCGAGCGCCTCTCTTCCCCCCTCCCCCACCT
ABCC6P2  CTTGTTGCAGGGGGAAGAGGGAACTCTGGAGGTGTCACTGAACTTTCAGGGGTTCCGGGACCCCCCAACCCGGTGCCCGTCCCAGCTCCCCGAGCGCCTCTCTTCCCCCATCCCCCACCT

         P300   GATA-2   Sp1   Sp1
ABCC6    CGCCTGTTTTCACCTCCCGTGGCCTCACTCCCGCCGCGCAGCTGGACCTTGCCCGGGGCCTCCCGATCCCGGAGCTCGAATCCCAGCCGGACCAGCCCAGCCCGACCAGCCCAGCCCCGC   -59
ABCC6P1  CGCCTGTTTTCACCTCCCGTGGCCTCACTCCCGCCGCGCAGCTGGACCTTGCCCGGGGCCTCCCGATCCCGGAGCTCGAATCCCAGCCGGACCAGCCTAGCCCGACCAGCCCAGCCCCGC
ABCC6P2  CGCCTGTTTTCACCTCCCGTGGCCTCACTCCCGCCGCGCAGCTGGACCTTGCCCGGGGCCTCCCGATCCTGGAGCTCGAATCCCAGCCGGACCAGCCCAGCCCGACCAGCCCAGCCCCGC

         GATA-2   ↱+1
ABCC6    CTCTTCTCCGGCAGGATCGCGGCCGAGCAGTCTGCCCAGAGACTTAGCGACAGACAGACGCTGGGACCCACGACGACAGAAGGCGCCGATGGCCGCGCCT   +42
ABCC6P1  CTCTTCTCCGGCAGGATCGCGGCCGAGCAGTCTGCCCAGAGACTTAGCGACAGACAGACGCTGGGACCCACGACGACAGAAGGCGCCGATGGCCGCGCC-
ABCC6P2  CTCTTCTCCGGCAGGATCGCGGCCGAGCAGTCTGCCCAGAGACTTAGCGACAGACAGACGCTGGGACCCACGACGACAGAAGGCGCCGATGGCCGCGCCT
```
