# Supplementary material for: The human ABC transporter pseudogene family: Evidence for transcription and gene-pseudogene interference
Source: BMC Genomics. 2008 Apr 11;9:165. doi: 10.1186/1471-2164-9-165 (PMC2329642; doi:10.1186/1471-2164-9-165)
Supplement: Additional file 3 — Similarities (%) between the members of the ABCB10 gene-pseudogene cluster. [file 1471-2164-9-165-S3.doc]

**Additional File 3. Similarities (%) between the members of the ABCB10 gene-pseudogene cluster.**

|  | ABCB10 | ABCB10P1 | ABCB10P2 | ABCB10P3 |
| --- | --- | --- | --- | --- |
| ABCB10 | 100% | 93.5% | 93.4% | 93.4% |
| ABCB10P1 |  | 100% | 99.4 | 99.4% |
| ABCB10P2 |  |  | 100% | 99.7% |
| ABCB10P3 |  |  |  | 100% |
